# Accelerating Visual Anticipation in Sport Through Temporal Occlusion Training: A Meta-Analysis

**DOI:** 10.1007/s40279-024-02073-6

**Published:** 2024-08-05

**Authors:** Sean Müller, Khaya Morris-Binelli, David Z. Hambrick, Brooke N. Macnamara

**Affiliations:** 1 Centre for Smart Analytics, Federation University Australia, University Drive, Ballarat, 3350 Australia; 2 School of Health Sciences, The University of Notre Dame Australia, Fremantle, Australia; 3 Department of Psychology, Michigan State University, Michigan, USA; 4 Department of Psychological Sciences, Case Western Reserve University, Cleveland, USA

## Abstract

**Background:**

The video-based temporal occlusion paradigm has been consistently used in visual anticipation sport research.

**Objective:**

This meta-analysis investigated the magnitude to which video-based temporal occlusion training could improve anticipation skill with transfer to representative laboratory and field tasks.

**Methods:**

As there are considerably fewer anticipation training than performance studies, the meta-analysis included 12 intervention studies with 25 effect sizes where video simulation and/or field-based tests were used. The Downs and Black checklist adapted for sports science research was used to assess methodological quality of the included studies. Decision time and accuracy of anticipation were the outcome measures because both are relevant to sports skills. The changes in these measures between experimental and control groups from baseline to the transfer test context were used to calculate the magnitude of the training effect.

**Results:**

Findings revealed a significant training effect, including a large meta-analytic effect size, and no difference in training benefit across video and field-based transfer tests. Publication bias analyses were inconclusive, likely due to the small number of available studies.

**Conclusions:**

These findings are evidence that the temporal occlusion paradigm is an effective method to improve visual anticipation skill across representative perceptual and perceptual-motor transfer tests. The theoretical implication based upon the two-stage model of visual anticipation is that temporal occlusion training can improve use of early information for body positioning by the performer, which could in turn lead to improved execution of the skill goal.

**Supplementary Information:**

The online version contains supplementary material available at 10.1007/s40279-024-02073-6.

## Key Points

**Table:** 

Large-effect-size improvements in visual anticipation were found from video-based temporal occlusion training that transferred to laboratory and field-based tests.
Temporal occlusion training is a cost-effective and portable technique that can be easily implemented to accelerate visual anticipation skill across a variety of sports.

## Introduction

Visual anticipation is a critical skill required for expert performance in high-speed sports [1]. It is needed due to the extreme temporal constraints encountered in sports, such as baseball and cricket batting or the return of serve in tennis, where combined choice reaction and movement times exceed the ball travel time to achieve the skill goal [2]. Visual anticipation is defined as the capability to use contextual information, such as the pitch count and field placings, as well as opponent kinematic information, such as wrist position, in sports such as baseball and cricket batting to predict object flight [3]. Several studies have reported that experts have superior anticipation skill compared with lesser skilled players on the basis of their capability to use pre-object flight (known as advance cue) information [4]. Fewer studies have investigated anticipation training, but these studies are crucial to determine whether performance can be accelerated and transferred to different contexts [5].

The temporal occlusion method has been consistently used to discriminate anticipation capability between expert and lesser-skilled performers, but also to train anticipation skill [6, 7]. Typically, in the discrimination approach, sports-specific footage of an opponent such as a pitcher or bowler executing different skill types is filmed, with the footage edited to place a black video frame prior to object flight [8]. This is done to control the duration of available visual information to advance cues, with the purpose of understanding the timing of visual information required for anticipation. A series of temporal occlusion video clips of different skill types are then assembled into a randomized sequence and displayed on a laptop or a projection screen. Performers, such as baseball or cricket batters, are required to watch the video clips and predict object flight using either a verbal, written, or simulated motor response [2, 8]. When temporal occlusion is used for training, a video clip is shown to the performer, and after a prediction is made, feedback is provided through an non-occluded replay of the previously viewed video clip [9]. Accordingly, the performer is given feedback on each training trial of the object outcome relative to the prediction they made. Therefore, the temporal occlusion method has dual utility for assessment and training of visual anticipation.

Studies that have investigated whether visual anticipation skill can be improved have compared a variety of instructional approaches. Some example studies have reported that video-based point-light displays [10], video-based temporal occlusion training [9], or video-based temporal occlusion training under simulated performance anxiety [11] improves anticipation skill. The critical design feature, however, is that such intervention studies have tightly controlled the duration (or timing) of visual information through the temporal occlusion paradigm (e.g., [9]). This is crucial to establish whether improvements gained relate to pickup of advance cue information rather than object flight information. Through tight control of visual information, an initial step has been made to demonstrate that anticipation can be improved from baseline assessment to enhanced pickup of advance information in a re-test assessment [4]. The more important question is whether temporal occlusion training can improve anticipation so that it transfers to a different context(s) to the one experienced during the training phase [5].

Transfer can be conceptualized as the influence of experience in one context to another context [12]. Contextual variation can refer to different stimuli, such as opponents presented in a video simulation or field test, as well as an action response required in a field test [5]. Investigation of whether transfer occurs to a field context of a sport is challenging because several opponents (e.g., baseball pitchers) are required to repeatedly execute skills (e.g., fastball) so that a suitable number of trials are presented to performers (e.g., baseball batter) to ensure valid and reliable performance measures. In addition, due to unpredictable weather conditions and exposure of expensive equipment to weather conditions, there are further limitations to conducting intervention studies in outdoor field settings. Together, these factors can limit the number of test trials that can be implemented, thus compromising use of large participant sample sizes, as well as implementation of field-based tests [13]. Studies that have included transfer contexts in their design through different opponents or a motor response have reported benefits based upon temporal occlusion training to video- and field-based tests [9, 10]. This indicates that video-based temporal occlusion training can improve anticipation beyond the training stimulus and, importantly, to motor action in field settings. However, the magnitude of transfer benefit from temporal occlusion training has not been quantified in the literature.

The relevance of anticipation and its training to high-speed sports skills has been conceptualized in a two-stage model [4]. In stage one, advance cue information involving game contextual and opponent kinematic information from the opponent can be used by expert performers to position their lower body for object interception. For example, in cricket batting this refers to use of bowler ball release location to judge whether the ball will be full or short and respond with a forward or backward foot movement. In tennis, observation of server kinematics until the point of racquet–ball contact would be used for lower-body positioning to the forehand or backhand side of the court. In stage two, object flight information is used to guide the striking implement to intercept the object. For example, in cricket batting and the return of serve in tennis, this refers to the use of ball flight and bounce information to fine-tune bat and racquet position to intercept the ball, respectively. Because video-based training has been reported to improve anticipation skill, it has the capacity to improve pickup of advance cues in stage one, which will allow more time to be able to position the body for interception in stage two.

The purpose of this meta-analysis was to determine the magnitude of anticipation transfer improvement that has been reported from video-based temporal occlusion training. We focused specifically on studies that included a video and/or field-based transfer test and compared performance change from baseline assessment to the transfer contexts. Comparison was also made as to whether transfer differed between video- and field-based tests. This is crucial for two reasons. First, from a theoretical perspective, meta-analytic evidence of training transfer will provide information as to whether video-based perceptual training contributes to pickup of advance information in perceptual and perceptual-motor tasks. Second, from a practical perspective, quantification of the magnitude of training transfer allows skill acquisition specialists, coaches, and athletes to make better-informed decisions about the impact of temporal occlusion training to accelerate skill acquisition.

## Methods

**Fig. 1:**
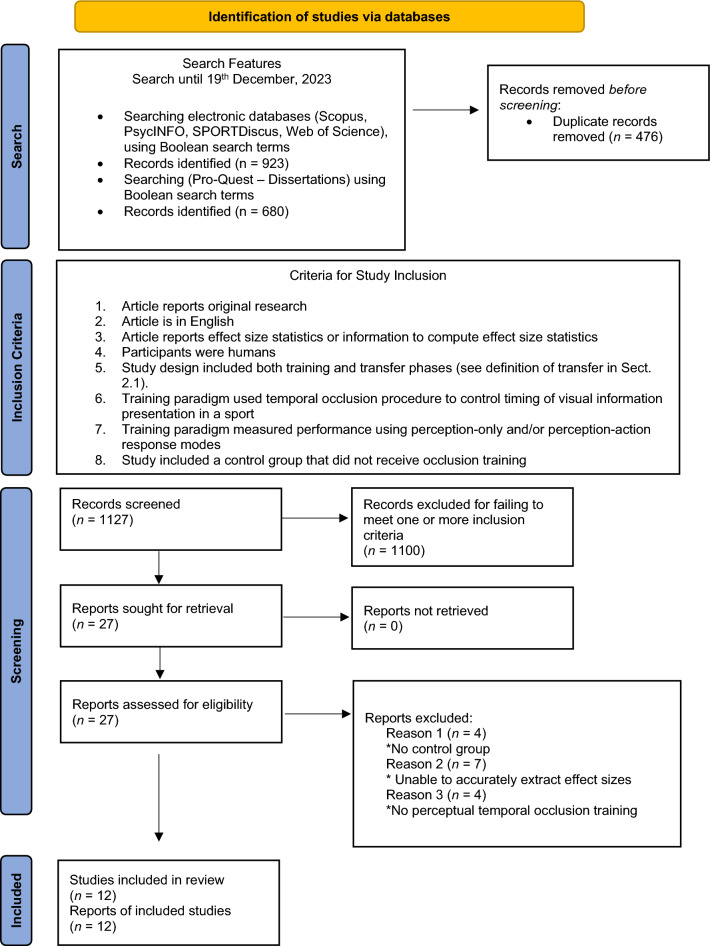
Preferred reporting format for systematic reviews and meta-analyses (PRISMA)

This meta-analysis followed the Preferred Reporting Items for Systematic reviews and Meta-Analyses (PRISMA) guidelines as outlined by Page et al. [14]. Accordingly, a flowchart is provided that outlines the steps undertaken for the meta-analysis search and screening process (Fig. 1). The search included studies that were published until 19 December 2023. The following search terms were used in the Scopus, PsycINFO, SPORTDiscus, and Web of Science databases: Sport* AND anticipation train* AND transfer*(1), Sport* AND visual-perceptual train* AND transfer*(2), Sport* AND perceptual train* AND transfer*(3), Sport* AND temporal occlusion train* AND transfer*(4), Sport* AND perceptual-cognitive skill train* AND transfer*(5), Sport* AND temporal occlusion AND observation train* AND motor training (6), Sport* AND temporal* AND occlusion* AND train* (7), Motor AND temporal* AND occlude* AND train*(8), Sport* AND decision-making AND train* AND transfer*(9). The following search terms were used in Pro-Quest—Dissertations: Sport* AND anticipat* AND occlu* AND train* AND transfer AND expert* AND (la.exact(“ENG”) AND subt.exact(“kinesiology” OR “psychology” OR “cognitive psychology”) (1), Sport* AND percept* AND cognit* AND occlu* AND train* AND transfer AND expert* AND (la.exact(“ENG”) AND subt.exact(“cognitive psychology” OR “psychology” OR “kinesiology”) (2).

### Inclusion and Exclusion Criteria

The inclusion and exclusion criteria for this meta-analysis are reported in the second box in Fig. 1. In relation to criteria points 5 and 6, video-based temporal occlusion training needed to have been included in the training phase with transfer assessment to different contexts. As mentioned earlier, transfer was conceptualized as contextual variation in terms of: (i) stimuli in the video-based pre- and post-tests compared with the training phase, (ii) opponents faced in the video-based tests compared with the training phase, or (iii) video-based training compared with a field-based perception–action test. For example, in Brenton et al. [10] temporal occlusion training was delivered through point-light displays of a bowler, whilst the transfer test included video temporal occlusion of a bowler not used in the pre–post-tests or intervention phases. In another example, Gabbett et al. [19] used video temporal occlusion training that included patterns of soccer match play, whilst the transfer test involved a pre–post-test of field small-sided games. In relation to inclusion criterion 7, “perception-only” refers to a video assessment task where perception is not coupled to action, whilst “perception–action” refers to a field-based assessment task where perception is coupled to action.

### Sample

Due to the small number of temporal occlusion training studies, and based upon our selection criteria, 12 studies including 25 effect sizes were suitable for inclusion in the meta-analysis reported in this paper. All studies were published papers and from a diverse range of sports including tennis, cricket, badminton, basketball, softball, soccer, rugby league, field-hockey, Australian Rules football, darts, and handball. These sports include skills that can be classified as open (e.g., cricket) or closed (e.g., darts), which require anticipation based upon opponent information or performer execution of movements, respectively, to achieve the skill goal. Participants in the studies that were included in the meta-analysis (*N* = 248) ranged from novices to highly skilled performers, with the latter (*n* = 107) including those who had participated in national or international level competition.

### Effect Size Data Acquisition and Meta-analytic Procedure

Three steps were undertaken to calculate standardized mean effect size (i.e., Cohen’s *d*) difference between an intervention and the control group for meta-analysis. First, the experimental groups’ measures (response accuracy and/or time) and tests (video and/or field) were identified for the selected studies (Table 1). Second, the pooled standard deviation from the pre-test for the intervention and control groups was calculated. Then, the pre-test-to-post-test mean difference was calculated for the intervention and control groups. Third, Cohen’s *d* effect size was calculated by subtracting the intervention group pre-post mean difference from the control group pre-post mean difference and dividing by the pre-test pooled standard deviation (see [15]).

**Table 1:** Overview of the studies included in the meta-analytic analysis including transfer test type, participant skill level, experimental group comparison and sample size, and outcome measure

Study	Test type	Participants	Groups compared	Measure
Alder et al., 2016 [11]	Field^a^	International badminton players	High-anxiety training (*n* = 10) versus control (*n* = 10)	Response accuracy
Gorman and Farrow, 2009 [16]	Field^a^	Skilled basketball players	Implicit training (*n* = 10) versus control (*n* = 10)	Response accuracy
Williams et al., 2003 [17]	Field^a^	Novice field hockey goalkeepers	Training (*n* = 8) versus control (*n* = 8)	Response accuracy and response time
Gabbett et al., 2007 [9]	Field	Elite softball players	Perceptual training (*n* = 9) versus control (*n* = 8)	Response accuracy
Williams et al., 2002 [7]	Field	Recreational tennis players	Guided discovery training (*n* = 8) versus control (*n* = 5)	Response accuracy and response time
Smeeton et al., 2005 [18]	Field	Intermediate tennis players	Guided discovery training (*n* = 10) versus control (*n* = 8)	Response accuracy and response time
Gabbett et al., 2008 [19]	Field^a^	Elite soccer players	Training (*n* = 8) versus control (*n* = 8)	Response accuracy (passing %)
Williams et al., 2002 [7]	Video^a^	Recreational tennis players	Guided discovery training (*n* = 8) versus control (*n* = 5)	Response accuracy and response time
Gorman & Farrow, 2009 [16]	Video^a^	Skilled basketball players	Implicit training (*n* = 10) versus control (*n* = 10)	Response accuracy
Smeeton et al., 2005 [18]	Video^a^	Intermediate tennis players	Guided discovery training (*n* = 10) versus control (*n* = 8)	Response accuracy and response time
Gabbett et al., 2007 [9]	Video^a^	Elite softball players	Perceptual training (*n* = 9) versus control (*n* = 8)	Response accuracy
Murgia et al., 2014 [20]	Video	Skilled soccer goalkeepers	Experimental (*n* = 13) versus control (*n* = 12)	Response accuracy
Williams et al., 2003 [17]	Video^a^	Novice field hockey goalkeepers	Training (*n* = 8) versus control (*n* = 8)	Response accuracy and response time
Mulligan et al., 2016 [21]	Video	Novice dart players	Perceptual training (none; *n* = 10) versus no practice (none; *n* = 10)	Response accuracy (dynamic stimuli)
Gabbett et al., 2008 [19]	Video^a^	Elite soccer players	Training (*n* = 8) versus control (*n* = 8)	Response accuracy (pattern prediction)
Brenton et al., 2019 [10]	Video	Skilled cricket batters	Visual-perceptual training (*n* = 13) versus control (*n* = 13)	Response accuracy
Alder et al., 2016 [11]	Video^a^	International badminton players	High-anxiety training (*n* = 10) versus control (*n* = 10)	Response accuracy
Alsharji & Wade, 2016 [22]	Video	Elite and skilled European handball goalkeepers	Perceptual training (*n* = 14) versus control (*n* = 14)	Response accuracy
Lorains et al., 2013 [23]	Video	Elite Australian Rules football players	Normal vision training (*n* = 15) versus control (*n* = 14)	Response accuracy

^a^Study included both field- and video-based transfer tests

For studies where the necessary mean and standard deviation information were not reported, we contacted the corresponding author for each of the selected studies via email with a request to provide descriptive data (means and standard deviations) for the experimental groups. Several authors either did not reply or were unable to locate the data because the study was more than 10 years old. For those studies where descriptive data were not provided by the authors, GetData Graph Digitizer Software (version 2.26.0.20) was used to manually extract mean and standard deviation/standard error values. These data were used to calculate standardized mean effect sizes.

An overall meta-analytic analysis was conducted examining standardized mean differences between trained and control groups on performance transfer. Performance transfer was operationalized as response accuracy and/or response time. The rationale for combining these two measures was because high-speed sports require both a high degree of accuracy and timing of responses [4]. The sign of response time effect sizes was changed to reflect the same interpretation as response accuracy effect sizes: positive effect sizes indicated transfer benefit from temporal occlusion training. Data were submitted to a random effects meta-analysis using the Comprehensive Meta-Analysis (Version 2) statistical software (see [15]). In addition, we tested whether the transfer test type (video versus field) significantly moderated the size of the effect. Publication bias was assessed using Egger’s regression as well as Duval and Tweedie’s trim and fill analysis. An alpha level of 0.05 was set for all analyses.

## Results

### Meta-analytic Analysis

We subjected the effect sizes to a random-effects meta-analysis to determine the overall effect of temporal occlusion training transfer and whether the size of the effect depended on the type of transfer test (video versus field). Table 1 outlines details of the studies included in the meta-analysis in relation to skill level of participants, test types, experimental groups compared, and the respective measures analyzed.

**Fig. 2:**
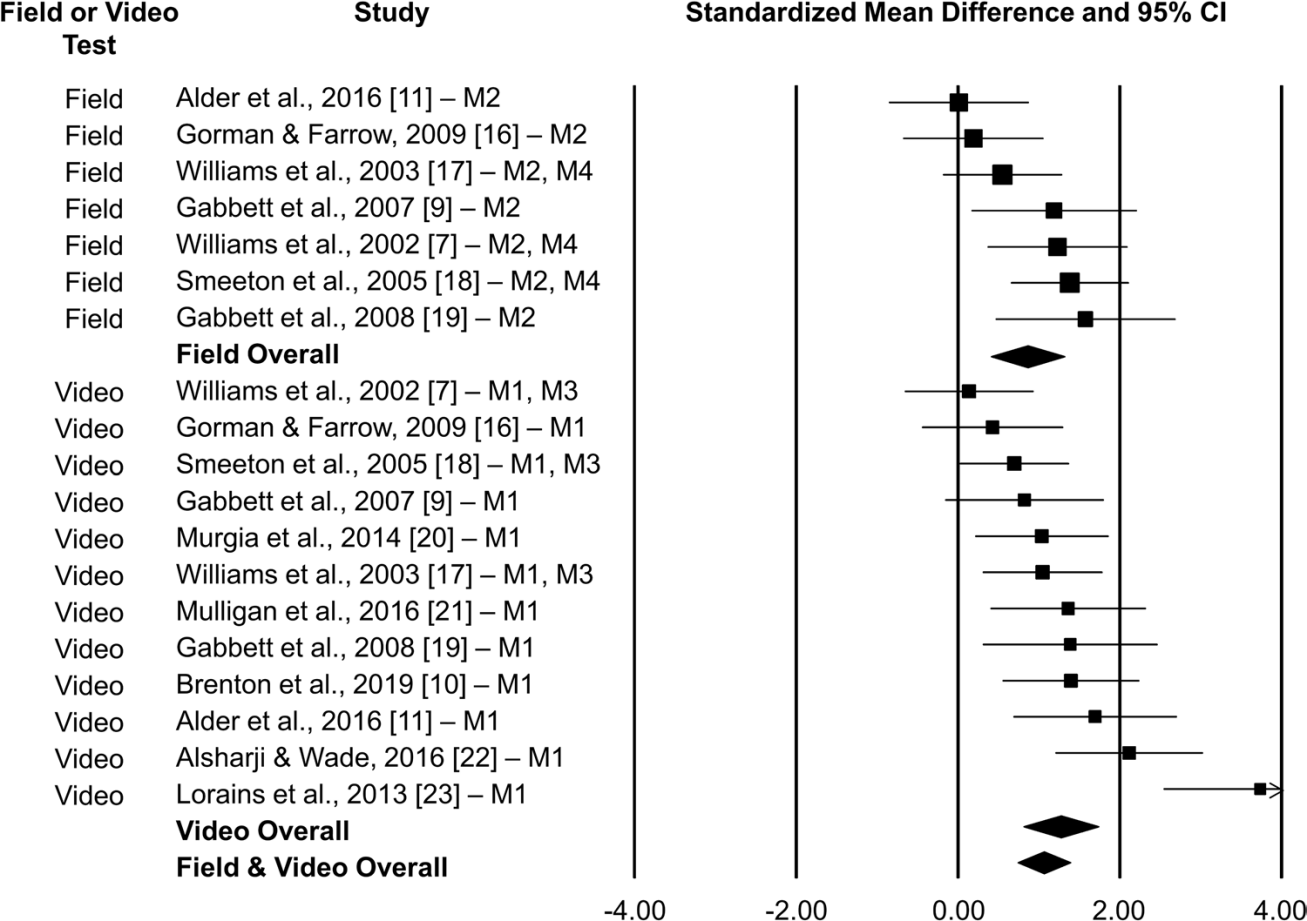
Forest plot of field and video tests and overall meta-analysis effect sizes with 95% confidence interval. M1 and M3 refer to video test response accuracy and timing measures, respectively, whilst M2 and M4 refer to field test response accuracy and timing measures, respectively. Positive effect sizes indicate improvement in anticipation relative to the control group

We first checked for outliers by examining whether any study’s residual was greater than three standard deviations from the mean within the transfer test type. No outliers were identified. Figure 2 graphs each of the selected studies’ effect sizes and the overall meta-analytic effect size. The overall analysis found a significant large effect for temporal occlusion training transfer (*Q*(11), 31.40; *p* < 0.001; $\overline{d }$ = 1.21; 95% confidence interval (CI) [0.83, 1.59]).

Heterogeneity was moderate (*τ*^2^ = 0.27; *I*^2^ = 64.97). We investigated whether heterogeneity was explained by the type of transfer test. Effects did not significantly differ between video tests ($\overline{d }$ = 1.26; 95% CI [0.80, 1.72]; *p* < 0.001) and field tests ($\overline{d }$ = 0.85; 95% CI [0.40, 1.30]; *p* < 0.001; *Q*(1), 1.55; *p* = 0.211).

Inspection of the effect sizes across studies indicated that the magnitude of improvement was not necessarily due to greater improvements for novice or lower-skilled participants. For example, studies by Alder et al. [11], Alsharji and Wade [22], Brenton et al. [10], Gabbett et al. [9], and Gabbett et al. [19] included skilled to highly skilled players in whom the magnitude of improvement in anticipation skill was descriptively large (mean *d* = 0.85, 2.12, 1.40, 1.00, and 1.48, respectively).

### Study Quality Assessment

An adapted version of the Downs and Black [24] scale, which has previously been deemed relevant to sports science research (see [25]) was used to rate the methodological quality of the studies included in this meta-analysis. The first (S.M.) and second (K.M.B.) authors rated the studies, and any disagreement was resolved through discussion. Study quality ranged between 9 and 11 out of a scale of 14 (Supplementary Table S1).

### Sensitivity Analyses

To ensure that observed effects were not unduly influenced by a single study, we conducted leave-one-out analyses. Leave-one-out analyses perform multiple meta-analytic models, each time excluding a single study. This process allows researchers to observe how much each study influences the aggregate results. We conducted these analyses for the overall effect, video transfer tests, for field-based transfer tests, measures of accuracy, and measures of decision speed.

The leave-one-out analyses yielded overall effects from $\overline{d }$ = 1.01 and 95% CI [0.76, 1.26] to $\overline{d }$ = 1.29 and 95% CI [0.90, 1.68], all with *p* < 0.001. We further examined leave-one-out analyses for only video-based tests ($\overline{d }$ = 1.06 and 95% CI [0.72, 1.39] to $\overline{d }$ = 1.36 and 95% CI [0.91, 1.82], all with *p* < 0.001), only field-based tests ($\overline{d }$ = 0.73 and 95% CI [0.25, 1.22] to $\overline{d }$ = 0.98, and 95% CI [0.56, 1.41], all with *p* < 0.003), only measures of accuracy ($\overline{d }$ = 0.87 and 95% CI [0.47, 1.26] to $\overline{d }$ = 1.17 and 95% CI [0.65, 1.69], all with *p* < 0.001), and only measures of decision speed ($\overline{d }$ = 1.50 and 95% CI [0.97, 2.03] to $\overline{d }$ = 1.75 and 95% CI [1.26, 2.23], all with *p* < 0.001). In 44 of the 46 cases, the 95% confidence interval included the overall observed effect with all studies of $\overline{d }$ = 1.21. In the remaining two cases, both bounds of the 95% confidence interval were larger than 1.21. Taken together, these results suggest that the effect of temporal occlusion training on performance is robust.

### Publication Bias

Multiple publication bias analyses were conducted to determine whether our results were influenced by potential publication bias of available studies. Each type of analysis has strengths and weaknesses, and so multiple analyses should be conducted.

The first type of publication bias analysis we conducted was Egger’s regression intercept [26]. Egger’s regression intercept quantifies funnel plots to assess whether small studies with lower-than-average effect sizes appear to be under-represented. The Egger’s regression intercept results suggested the presence of publication bias [intercept = 3.58; standard error (SE) = 1.48; *p* (1 − tailed recommend) = 0.018].

The second type of publication bias analysis we conducted was Duval and Tweedie’s trim and fill analysis [27]. This analysis quantifies funnel plots to assess how many smaller-than-average effect sizes appear to be missing and imputes a new overall effect size if these studies were not missing. Duval and Tweedie’s trim and fill suggested that no studies with smaller-than-average effects were missing from our analysis.

These inconsistent publication bias analysis results are likely due to the small number of studies available to analyze, which leads to imprecise estimates. On the basis of this variability, we cannot conclude whether publication bias exists in the literature or whether, and to what extent, our results are influenced by its presence.

## Discussion

The purpose of this meta-analysis was to quantify the magnitude of video-based temporal occlusion training transfer to video and field-based contexts. Understanding whether anticipation can be improved is crucial because it is a vital skill to deal with the high time constraints in several sports and, therefore, for the facilitation of superior performance. Furthermore, knowing whether this crucial skill can be improved to the extent where there is benefit to perception-only (video) and perception–action (field) tasks has important theoretical and practical implications for researchers, practitioners, and athletes. Study design quality assessment indicated that the included studies had good methodological rigor. Further, sensitivity analyses revealed that no single study was unduly influencing the meta-analytic outcomes. Therefore, despite inconclusive publication bias analyses, it was possible to consider the relevance of temporal occlusion training from theoretical and applied perspectives.

The overall large effect size from video-based temporal occlusion training suggests that substantial improvements in anticipation skill are possible. To contextualize this training benefit, it is important to consider that our sample of selected studies included participants that ranged from novices (e.g. [21] to highly skilled athletes (e.g. [19]). The skilled athletes included those who had participated at national and international levels or those who were part of an elite squad (e.g., [10, 19], respectively). Therefore, our results not only are driven by a greater magnitude of improvement in lower-skilled performers but also suggest that highly skilled athletes can benefit from this type of training.

The magnitude of video-based temporal occlusion training improvement did not differ between video (perception) and field (perception–action) transfer contexts. This is an important finding because it suggests that video-based perceptual training similarly benefits perception- and perception–action-based tasks. The reasons for this are twofold. First, from a behavioral perspective, all the studies in our meta-analysis included representative visual-perceptual information of an opponent such as what a performer sees in the real world [28]. Accordingly, the benefits of the video-based temporal occlusion training mapped across perceptual and action responses is likely due to representative visual-perceptual information design [29]. It is relevant to consider here that none of the studies included contextual information. Despite previous studies that have reported contextual information can be utilized to enhance anticipation (e.g., [37]), recent research has reported that overreliance on such information can impede the use of kinematic information for anticipation (e.g., [38]). Second, from a neurophysiological perspective, evidence indicates that sensorimotor regions of the brain are engaged both in perceptual and perceptual-motor anticipation tasks [30]. As a result, representative video-based perceptual training should engage sensory and motor regions in the brain that will map across perceptual and motor tasks. This is in contrast to literature that argues that visual perceptual information must be coupled with a sport-specific motor response for meaningful skill improvement to occur (e.g., [31]).

The findings of this meta-analysis have important implications for the two-stage model of anticipation by Morris-Binelli and Müller [4]. First, it is possible to improve pickup of advance cues in stage one, which is used for body positioning. This is critical because inability to integrate contextual and kinematic cues can cause a delay or reduced accuracy in motor responses such as foot movements in cricket, baseball, and softball batting, or stepping in the return of serve in tennis [32, 33]. These errors can cascade into stage two of the model to cause timing or error in positioning the implement such as a bat, racquet, hand, or foot to intercept an object [32, 33]. Therefore, video-based temporal occlusion training can guide attention to the pickup of advance information, which may better enable integration with object flight information to achieve skill goal.

Second, the future research directions section of the original two-stage model paper on anticipation [34] outlined a need for further studies to demonstrate whether anticipation skill can be improved. Since the original two-stage model paper was published, five temporal occlusion training studies have been conducted on the basis of our selection criteria, with the magnitude of the anticipation improvement in their, and some other, studies captured in this meta-analysis. Underpinned by tightly controlled stimulus presentation of temporal occlusion, it appears a variety of instructional methods such as point-light displays [10], two-dimensional video [19], above real time video [23], and anxiety-based perceptual training [11] may improve anticipation, particularly in stage one. The benefits of improved anticipation in relation to the model present several practical implications.

Video-based temporal occlusion training is not an expensive method and can be easily incorporated into transportable software applications or virtual reality headsets [35]. For example, software can be used to edit footage of sports performers, such as baseball and softball pitchers as well as tennis servers. Thereafter, the temporal occlusion training technique can be applied. Varying degrees of contextual information, opponent kinematics, and action type challenges can be designed, where training can be done in a quiet room at a sports facility, whilst travelling for competition, or at home (e.g., [19]). Incorporation of temporal occlusion training with routine practice can also provide a means to overcome limitations to securing opponents to practice against, as well as minimize physical loading during actual practice that can increase risk of injury across a variety of sports [36]. This presents flexible options for coaches and sports medicine support staff to train anticipation. For the practical and technical implications of temporal occlusion training, see the audio-visual recording in the Electronic Supplementary Material 2.

## Conclusion

This meta-analysis results suggest that video-based temporal occlusion training can provide considerable improvement to anticipation skill in sport. Benefits to performance were limited not only to video-based tasks that are much easier to administer but also to field-based tasks that are much more challenging to implement and are closer to the competition setting. This provides justification for the use of temporal occlusion training to improve anticipation across the skill continuum in sport. A potential limitation of this meta-analysis is that there were only 12 studies. It needs to be considered, however, that we used strict selection criteria and training studies that include longitudinal designs that are challenging to implement, and therefore, fewer exist. With a larger pool of studies, a future meta-analysis may be able to include a variety of moderator variables such as length of training and participant skill level in addition to transfer test type. Additionally, future research should compare the magnitude of learning transfer from temporal occlusion training across point-light display, two-dimensional video, above real time video, and anxiety-based manipulations. This will provide guidance on how these manipulations can be incorporated with temporal occlusion training to facilitate transfer to the field setting. Nonetheless, this meta-analysis has reported that, by examining studies that tightly controlled the duration of visual information through the temporal occlusion method, valuable improvements may be made to the critical skill of visual anticipation.

## Supplementary Information

Below is the link to the electronic supplementary material.

Supplementary file1 (DOCX 20 KB)

Supplementary file2 (MP4 4473 KB)

## References

[1] Hodges NJ, Wyder-Hodge PA, Hetherington S, Baker J, Besler Z, Spering M. Topical review: perceptual-cognitive skills, methods, and skill-based comparisons in interceptive sports. Optom Vis Sci. 2021. 10.1097/opx.0000000000001727. 34328450 10.1097/OPX.0000000000001727

[2] Taliep MS, Mhlonngo N, Draper CE, Rust R, Gray J. The progressive use of visual cues in skilled adolescent cricket batters. Appl Cogn Psychol. 2023. 10.1002/acp.4109.

[3] Runswick OR, Roca A, Williams AM, McRobert AP, North JS. Why do bad balls get wickets? The role of congruent and incongruent information in anticipation. J Sport Sci. 2019. 10.1080/02640414.2018.1514165. 10.1080/02640414.2018.1514165 30132402

[4] Morris-Binelli K, Müller S. Advancements to the understanding of expert visual anticipation skill in striking sports. Can J Behav Sci. 2017. 10.1037/cbs0000079.

[5] Müller S, Rosalie SM. Transfer of expert visual-perceptual-motor skill in sport. In: Williams AM, Jackson CR, editors. Anticipation and decision making in sport. Oxon: Routledge; 2019. p. 375–93.

[6] Jones CM, Miles TR. Use of advance cues in predicting the flight of a lawn tennis ball. J Hum Mov Stud. 1978;4(4):231–5.

[7] Williams AM, Ward P, Knowles JM, Smeeton NJ. Anticipation skill in a real-world task: measurement, training, and transfer in tennis. J Exp Psychol Appl. 2002;8(4):259–70. 12570100 10.1037//1076-898x.8.4.259

[8] Müller S, Abernethy B, Farrow D. How do world-class cricket batsmen anticipate a bowler’s intention? Q J Exp Psychol. 2006. 10.1080/02643290600576595. 10.1080/02643290600576595 17095494

[9] Gabbett T, Rubinoff M, Thorburn L, Farrow D. Testing and training anticipation skills in softball fielders. Int J Sport Sci Coach. 2007. 10.1260/174795407780367159.

[10] Brenton J, Müller S, Dempsey A. Visual-perceptual training with acquisition of the observed motor pattern contributes to greater improvement of visual anticipation. J Exp Psychol Appl. 2019. 10.1037/xap0000208. 30688501 10.1037/xap0000208

[11] Alder D, Ford PR, Causer J, Williams AM. The effects of high- and low-anxiety training on the anticipation judgments of elite performers. J Sport Exerc Psychol. 2016. 10.1123/jsep.2015-0145. 27018561 10.1123/jsep.2015-0145

[12] Magill RA, Anderson D. Motor learning and control: concepts and applications. 12th ed. New York: McGraw Hill; 2021.

[13] Müller S, Brenton J, Rosalie SM. Methodological considerations for investigating expert interceptive skill in in situ settings. Sport Exerc Perform Psychol. 2015. 10.1037/spy0000044.

[14] Page MJ, McKenzie JE, Bossuyt PM, Boutron I, Hoffmann TC, Mulrow CD, et al. The PRISMA 2020 statement: An updated guideline for reporting systematic reviews. Syst Rev. 2021. 10.1186/s13643-021-01626-4. 10.1186/s13643-021-01626-4 PMC8008539 33781348

[15] Güllich A, Macnamara BN, Hambrick DZ. What makes a champion? Early multidisciplinary practice, not early specialization, predicts world-class performance. Perspect Psychol Sci. 2022. 10.1177/1745691620974772. 34260336 10.1177/1745691620974772

[16] Gorman AD, Farrow D. Perceptual training using explicit and implicit instructional techniques: does it benefit skilled performers? Int J Sports Sci Coach. 2009. 10.1260/174795409788549526.

[17] Williams AM, Ward P, Chapman C. Training perceptual skill in field hockey: is there transfer from the laboratory to the field? Res Q Exerc Sport. 2003. 10.1080/02701367.2003.10609068. 12659480 10.1080/02701367.2003.10609068

[18] Smeeton NJ, Williams AM, Hodges NJ, Ward P. The relative effectiveness of various instructional approaches in developing anticipation skill. J Exp Psychol Appl. 2005. 10.1037/1076-898X.11.2.98. 15998182 10.1037/1076-898X.11.2.98

[19] Gabbett TJ, Carius J, Mulvey M. Does improved decision-making ability reduce the physiological demands of game-based activities in field sport athletes? J Strength Cond Res. 2008. 10.1519/JSC.0b013e3181887f34. 18978606 10.1519/JSC.0b013e3181887f34

[20] Murgia M, Sors F, Muroni AF, Santoro I, Prpic V, Galmonte A, et al. Using perceptual home-training to improve anticipation skills of soccer goalkeepers. Psychol Sport Exerc. 2014. 10.1016/j.psychsport.2014.07.009.

[21] Mulligan D, Lohse KR, Hodges NJ. Evidence for dual mechanisms of action prediction dependent on acquired visual-motor experiences. J Exp Psychol Hum Percept Perform. 2016. 10.1037/xhp0000241. 27280709 10.1037/xhp0000241

[22] Alsharji KE, Wade MG. Perceptual training effects on anticipation of direct and deceptive 7-m throws in handball. J Sport Sci. 2016. 10.1080/02640414.2015.1039463. 10.1080/02640414.2015.1039463 25917061

[23] Lorains M, Ball K, MacMahon C. An above real time training intervention for sport decision making. Psychol Sport Exerc. 2013. 10.1016/j.psychsport.2013.05.005.

[24] Downs SH, Black N. The feasibility of creating a checklist for the assessment of the methodological quality both of randomised and non-randomised studies of health care interventions. J Epidemiol Community Health. 1998. 10.1136/jech.52.6.377. 9764259 10.1136/jech.52.6.377 PMC1756728

[25] Thurlow F, Huynh M, Townshend A, McLaren SJ, James LP, Taylor JM, et al. The effects of repeated-sprint training on physical fitness and physiological adaptation in athletes: a systematic review and meta-analysis. Sport Med. 2023. 10.1007/s40279-023-01959-1. 10.1007/s40279-023-01959-1 38041768

[26] Egger M, Smith GD, Schneider M, Minder C. Bias in meta-analysis detected by a simple, graphical test. BMJ. 1997. 10.1136/bmj.315.7109.629. 9310563 10.1136/bmj.315.7109.629 PMC2127453

[27] Duval S, Tweedie R. Trim and fill: a simple funnel-plot-based method of testing and adjusting for publication bias in meta-analysis. Biometrics. 2000. 10.1111/j.0006-341x.2000.00455.x. 10877304 10.1111/j.0006-341x.2000.00455.x

[28] Araújo D, Davids K. Towards a theoretically-driven model of correspondence between behaviours in one context to another: Implications for studying sport performance. Int J Sport Psychol. 2015;46:745–57.

[29] Kalén A, Bisagno E, Musculus L, Raab M, Pérez-Ferreirós A, Williams AM, et al. The role of domain-specific and domain-general cognitive functions and skills in sports performance: a meta-analysis. Psychol Bull. 2021. 10.1037/bul0000355. 35404636 10.1037/bul0000355

[30] Aglioti SM, Cesari P, Romani M, Urgesi C. Action anticipation and motor resonance in elite basketball players. Nat Neurosci. 2008. 10.1038/nn.2182. 19160510 10.1038/nn.2182

[31] Davids K, Araújo D, Seifert L, Orth D. Expert performance in sport: an ecological dynamics perspective. In: Baker J, Farrow D, editors. Routledge handbook of sport expertise. Florence: Routledge; 2015. p. 130–44.

[32] Takamido R, Yokoyama K, Yamamoto Y. Effect of manipulating advanced kinematic information on hitting movement prediction, perception, and action. Res Q Exerc Sport. 2021. 10.1080/02701367.2020.1773375. 32852245 10.1080/02701367.2020.1773375

[33] Taliep MS, Mhlonngo N, Draper CE, Rust R, Gray J. The progressive use of visual cues in skilled adolescent cricket batters. Appl Cog Psychol. 2023. 10.1002/acp.4109.

[34] Müller S, Abernethy B. Expert anticipatory skill in striking sports: a review and a model. Res Q Exerc Sport. 2012. 10.5641/027013612800745059. 22808703 10.1080/02701367.2012.10599848

[35] Fadde PJ. Instructional design for accelerated macrocognitive expertise in the baseball workplace. Front Psychol. 2016. 10.3389/fpsyg.2016.00292. 26973581 10.3389/fpsyg.2016.00292 PMC4773639

[36] Nassis GP, Brito J, Figueiredo P, Gabbett TJ. Injury prevention training in football: let’s bring it to the real world. Br J Sport Med. 2019. 10.1136/bjsports-2018-100262. 10.1136/bjsports-2018-100262 30926629

[37] Runswick OR, Roca A, Williams AM, McRobert AP, North JS. The temporal integration of information during anticipation. Psychol Sport Exerc. 2018. 10.1016/j.psychsport.2018.05.001.

[38] Müller S, Brenton J, O’Grady M. Reliance upon contextual information can impede visual anticipation. Eur J Sport Sci. 2023. 10.1080/17461391.2022.2157337. 36481092 10.1080/17461391.2022.2157337

