# Supplementary material for: Accelerating Visual Anticipation in Sport Through Temporal Occlusion Training: A Meta-Analysis
Source: Sports Med. 2024 Aug 5;54(10):2597–606. doi: 10.1007/s40279-024-02073-6 (PMC11467115; doi:10.1007/s40279-024-02073-6)
Supplement: Supplementary file 1 — Supplementary file1 (DOCX 20 KB) [file 40279_2024_2073_MOESM1_ESM.docx]

**Supplementary Table S1** Modified Downs and Black scale outcomes for the reporting quality and risk of bias assessment

| Study | Item Number | | | | | | | | | | | | | | Total score (out of 14) |
| --- | --- | --- | --- | --- | --- | --- | --- | --- | --- | --- | --- | --- | --- | --- | --- |
|  | 1 | 2 | 3 | 6 | 7 | 10 | 12 | 15 | 16 | 18 | 20 | 22 | 23 | 25 |  |
| Alder et al., 2016 [11] | 1 | 1 | 1 | 1 | 1 | 1 | 0 | 0 | 0 | 1 | 1 | 1 | 1 | 1 | 11 |
| Gorman & Farrow, 2009 [16] | 1 | 1 | 1 | 1 | 1 | 1 | 0 | 0 | 0 | 1 | 1 | 1 | 0 | 0 | 9 |
| Williams et al., 2003 [17] | 1 | 1 | 1 | 1 | 1 | 0 | 0 | 0 | 0 | 1 | 1 | 1 | 1 | 1 | 10 |
| Gabbett et al., 2007 [9] | 1 | 1 | 1 | 1 | 1 | 1 | 0 | 0 | 0 | 1 | 1 | 1 | 1 | 0 | 10 |
| Williams et al., 2002 [7] | 1 | 1 | 1 | 1 | 1 | 0 | 0 | 0 | 0 | 1 | 1 | 1 | 1 | 1 | 10 |
| Smeeton et al., 2005 [18] | 1 | 1 | 1 | 1 | 1 | 0 | 0 | 0 | 0 | 1 | 1 | 1 | 0 | 1 | 9 |
| Gabbett et al., 2008 [19] | 1 | 1 | 1 | 1 | 1 | 0 | 0 | 1* | 0 | 1 | 1 | 1 | 0 | 1 | 10 |
| Murgia et al., 2014 [20] | 1 | 1 | 1 | 1 | 1 | 0 | 0 | 0 | 0 | 1 | 1 | 1 | 1 | 1 | 10 |
| Mulligan et al., 2016 [21] | 1 | 1 | 1 | 1 | 1 | 1 | 0 | 0 | 0 | 1 | 1 | 1 | 1 | 1 | 11 |
| Brenton et al., 2019 [10] | 1 | 1 | 1 | 1 | 1 | 1 | 0 | 0 | 0 | 1 | 1 | 1 | 1 | 1 | 11 |
| Alsharji & Wade, 2006 [22] | 1 | 1 | 1 | 1 | 1 | 1 | 0 | 0 | 0 | 1 | 1 | 1 | 1 | 1 | 11 |
| Lorains et al., 2013 [23] | 1 | 1 | 1 | 1 | 1 | 1 | 0 | 0 | 0 | 1 | 1 | 1 | 1 | 0 | 11 |

*Note.* 0 = no; 1 = yes. Item 1: clear aim/hypothesis; Item 2: outcome measures clearly described; Item 3: patient [athlete] characteristics clearly described; Item 6: main findings clearly described; Item 7: measures of random variability provided; Item 10: actual probability values reported; Item 12: participants prepared to participate representative of the entire population; Item 15: blinding of outcome measures; Item 16: analysis completed was planned; Item 18: appropriate statistics; Item 20: valid and reliable outcome measures; Item 22: participants recruited over the same period; Item 23: randomised; Item 25: adjustment made for confounding variables. *On-field decision-making outcome measure was blinded.
